# Genome-wide identification and expression analysis of the *TaRRA* gene family in wheat (*Triticum aestivum* L.)

**DOI:** 10.3389/fpls.2022.1006409

**Published:** 2022-08-30

**Authors:** Lijing Sun, Liangjie Lv, Jie Zhao, Mengyun Hu, Yelun Zhang, Yun Zhao, Xiaodong Tang, Peinan Wang, Qianying Li, Xiyong Chen, Hui Li, Yingjun Zhang

**Affiliations:** Laboratory of Crop Genetics and Breeding of Hebei, Institute of Cereal and Oil Crops, Hebei Academy of Agriculture and Forestry Sciences, Shijiazhuang, China

**Keywords:** type-A response regulators, gene family, wheat (*Triticum aestivum* L.), expression pattern, cytokinin, abiotic stress

## Abstract

Cytokinin is an important endogenous hormone in plants performing a wide spectrum of biological roles. The *type-A response regulators (RRAs)* are primary cytokinin response genes, which are important components of the cytokinin signaling pathway and are involved in the regulation of plant growth and development. By analysis of the whole genome sequence of wheat, we identified 20 genes encoding RRAs which were clustered into eight homologous groups. The gene structure, conserved motifs, chromosomal location, and *cis*-acting regulatory elements of the *TaRRAs* were analyzed. Quantitative real-time polymerase chain reaction (qRT-PCR) results showed that the expression levels of most of the *TaRRAs* increased rapidly on exogenous cytokinin application. Moreover, the *TaRRA* family members displayed different expression profiles under the stress treatments of drought, salt, cold, and heat. This study provides valuable insights into the *RRA* gene family in wheat and promotes the potential application of these genes in wheat genetic improvement.

## Introduction

Cytokinin is a vital phytohormone responsible for regulating numerous aspects of plant growth and development, including cell division and differentiation, apical dominance, leaf senescence, photomorphogenesis, fertility, and seed development (Werner and Schmülling, 2009; Hwang et al., 2012; Schaller et al., 2015; Wybouw and De Rybel, 2019). In addition, cytokinin plays an important role in plant response to many environmental stresses such as drought, salt, cold, and heat (Ha et al., 2012; Pavlů et al., 2018; Liu et al., 2020). Cytokinin signal transduction is mediated by a multistep two-component system (TCS) involving a His-Asp-His-Asp phosphorelay from histidine kinase receptors (HKs) and histidine-containing phosphotransfer proteins (HPs) to downstream response regulators (RRs) (Argueso et al., 2010; El-Showk et al., 2013; Kieber and Schaller, 2018).

The response regulators are classified into four distinct groups based on phylogenetic and conserved domain analysis, namely, type-A response regulators (RRAs), type-B response regulators (RRBs), type-C response regulators (RRCs), and circadian clock-related pseudo response regulators (PRRs) (Heyl et al., 2013), all with a conserved N-terminal receiver domain and a variable-length C-terminal. The C-terminal of RRAs is short and has yet to be assigned any specific function. RRBs have a large C-terminal extension containing Myb-like DNA binding, nuclear localization, and transcription activation domains (Sakai et al., 2000; Hosoda et al., 2002). The C-terminal of RRCs is also short; however, they are not grouped in the same class as RRAs. PRRs lack the highly conserved Asp residue required for phosphorylation and have a C-terminal including a CCT domain (CO, CO-LIKE, TOC1) (Makino et al., 2000; Schaller et al., 2008). RRBs are transcription factors that mediate the transcriptional response to cytokinin (Argyros et al., 2008). RRAs are transcriptionally induced in response to cytokinin *via* direct activation by RRBs and are responsible for repressing cytokinin signaling *via* a negative feedback loop (Hwang et al., 2012).

Many reports have established that RRAs play a critical role in plant growth and development. In *Arabidopsis*, 10 *RRAs* have been reported (*ARR3-ARR9* and *ARR15-ARR17*) (D’Agostino et al., 2000), of which *ARR3* and *ARR4* act redundantly in the determination of the circadian rhythm (Salomé et al., 2006). ARR4 interacts with phytochrome B, modulating red light signaling by stabilizing the active Pfr form of phytochrome B (Sweere et al., 2001; Mira-Rodado et al., 2007). *ARR7* negatively influences meristem size through regulation of its expression by WUSCHEL (Leibfried et al., 2005). Moreover, *ARR7*, together with *ARR15*, also participates in the cytokinin–auxin hormonal control of the shoot stem-cell niche (Zhao et al., 2010). In addition, *ARR16* has been reported to be involved in *Arabidopsis* seedling development *via* regulation of its expression by MYC2 (Srivastava et al., 2019).

Accumulating evidence indicates that RRAs are involved in abiotic stress responses. Dehydration stress transiently induces the expression of *ARR5, ARR7*, and *ARR15*, but reduces the expression of *ARR8* and *ARR17* (Kang et al., 2012). Also, the phosphorylation of ARR5 Ser residues by SnRK2s enhances its stability and plant drought tolerance (Huang et al., 2018). The expression of a variety of *RRAs*, including *ARR5, ARR6, ARR7*, and *ARR15*, are induced by cold (Jeon et al., 2010). However, *ARR-OE* plants (*ARR5-OE, ARR7-OE*, and *ARR15-OE*) and *arr* mutants (*arr5, arr6*, and *arr7*) show similar enhanced freezing tolerance, indicating that RRAs play a complex role in regulating cold stress response (Jeon et al., 2010; Shi et al., 2012). In rice, the expression of type-A *OsRR6* is induced by salt, dehydration, and low-temperature treatments (Jain et al., 2006), and overexpression of *OsRR6* enhances seedling drought and salinity tolerance (Bhaskar et al., 2021); whereas, *OsRR9* and *OsRR10* negatively regulate rice salinity tolerance (Wang et al., 2019). Recently, *ZmRR1*, a maize type-A RR, has been demonstrated to positively regulate maize chilling tolerance by modulating the expression of *ZmDREB1s* and *ZmCesAs*. The phosphorylation of ZmRR1 Ser residues by ZmMPK8 accelerates its degradation, thereby reducing the chilling tolerance (Zeng et al., 2021).

RRAs have been widely studied in *Arabidopsis* and rice, however, limited information is available for RRAs in wheat. The completion of the wheat whole genome sequence and further improvements of the wheat genome database have immensely contributed to decoding the wheat genome at the molecular level. In the present study, we systematically performed a genome-wide analysis of the wheat *RRA* gene family and investigated their gene structures, conserved motifs, chromosomal locations, *cis*-acting regulatory elements, and expression patterns in response to cytokinin treatment and various stresses. This work provides valuable information on the wheat *RRA* gene family and lays a foundation for further functional analysis of this gene family.

## Materials and methods

### Identification of *TaRRA* genes in wheat

Whole-genome and protein sequence data of wheat (IWGSC Assembly GCA_900519105.1) and the hidden Markov model (HMM) file for the response regulator receiver domain (PF00072) were downloaded from the EnsemblPlants database^1^ and Pfam database,^2^ respectively. A wheat-specific HMM file was established by the alignment of the response regulator receiver domain HMM file with the wheat protein sequences (E-value < 1e^–20^). The wheat-specific HMM file was then used as bait to search against the local reference genome database to identify candidate wheat RRs (E-value < 0.01). Redundant genes were removed, and the longest representative transcripts were selected for further analysis. The identified proteins were then submitted to Pfam (see text footnote 2), SMART,^3^ and NCBI conserved domains search tool^4^ to further check the receiver domain as well as other conserved domains. The protein sequence of RRs in wheat, *Arabidopsis*, and rice was used to carry out multiple sequence alignment using ClustalX 2.1 software (Larkin et al., 2007). The phylogenetic tree was established using MEGA 7.0 based on the neighbor-joining (NJ) method with 1,000 bootstrap replicates (Kumar et al., 2016), and RRs were named according to the standard nomenclature for the plant TCS elements (Heyl et al., 2013).

### Characterization of *TaRRAs*

Information about the *TaRRA* gene family, such as chromosomal localization, number of exons, and cDNA and protein length, was obtained from the EnsemblPlants. The protein sequence of TaRRAs was analyzed in the Expasy server^5^ to obtain the theoretical isoelectric point (PI) and molecular weight (MW).

### Gene structure and motif analysis of *TaRRAs*

The gene structure of *TaRRAs* was constructed by the gene structure display server (GSDS 2.0^6^) using the coding sequence (CDS) and corresponding genomic sequence retrieved from the EnsemblPlants database. Conserved motifs of TaRRAs were predicted using the Multiple Em for Motif Elicitation (MEME 5.4.1^7^), with the following parameters: maximum number of 10 motifs and optimum motif widths of 6-50 residues.

### Collinearity relationship of *TaRRAs*

The wheat genomic sequence and genome annotation files downloaded from the EnsemblPlants database were used to generate a graph of chromosomal location and collinearity relationship of *TaRRAs* by TBtools software (Chen et al., 2020). The synteny relationship of *RRAs* between wheat and rice was constructed using the Dual Systeny Plot for MCscanX.^8^

### *Cis*-acting elements analysis of *TaRRAs*

The promoter region, 1,500 bp upstream of the initiation code (ATG), of all of the *TaRRAs*, was obtained from the EnsemblPlants database and the *cis*-acting regulatory elements were predicted by PlantCARE.^9^

### Gene expression analysis of *TaRRAs*

The expression data in various tissues were downloaded from the WheatOmics 1.0 (Ma et al., 2021). The transcripts per million (TPM) values were used to create a heat map by using Heatmap (see text footnote 8).

Jimai325, a high-yielding and water-saving wheat variety cultivated by our lab, was used for qRT-PCR analysis. Ten-day-old hydroponically grown seedlings were exposed to 50 μM 6-BA, 20% PEG-6000 (drought stress), 200 mM NaCl (salt stress), 4°C (cold stress), or 40°C (heat stress) for 0, 1, 3, 6, 12, and 24 h, and samples were then collected. Total RNA was isolated using TRNzol Universal reagent (TIANGEN) according to the manufacturer’s instructions, and 1 μg of total RNA was used as the template for cDNA synthesis. Real-time PCR was subsequently performed to quantify the cDNA using SYBR Premix Ex Taq (TaKaRa) in a CFX96™ real-time PCR detection system (BIO-RAD). *TaActin* was used as an internal control to normalize all data. The primers used were listed in Supplementary Table 1.

## Results

### Identification and classification of *TaRRA* genes in wheat

**FIGURE 1:**
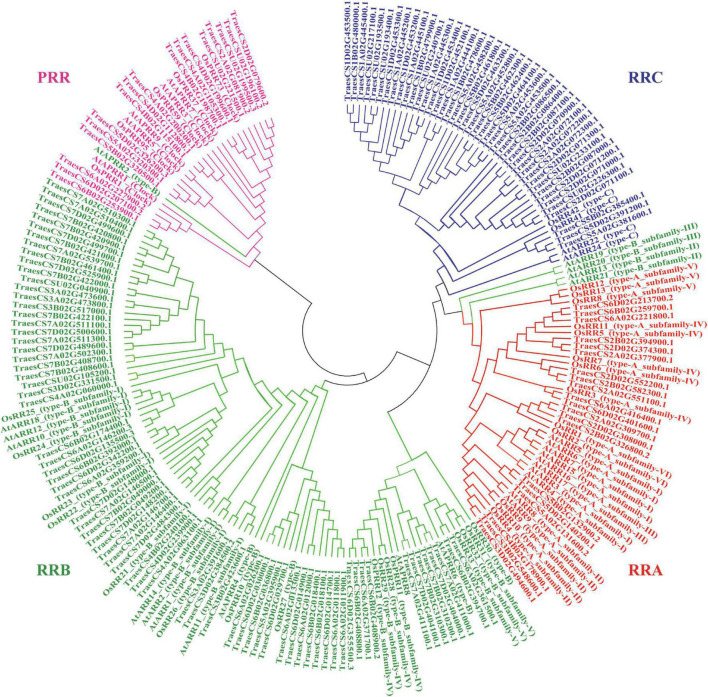
Phylogenetic analysis of RR proteins among the wheat, *Arabidopsis*, and rice. All RR proteins were allocated into four clades. Red, green, blue, and pink represent RRA, RRB, RRC, and PRR, respectively. The unrooted tree was established by the neighbor-joining method using the MEGA7.0 software with 1,000 bootstrap replicates.

The wheat-specific HMM file for the response regulator receiver domain was aligned with the whole protein sequences in wheat, and 151 non-redundant *TaRR* genes in wheat were identified after receiver domain confirmation. An unrooted phylogenetic tree was generated by using the conserved receiver domain and incorporating the well-established family members from *Arabidopsis* and rice for the subfamily classification of *TaRR*s (Figure 1; Heyl et al., 2013). To confirm the subfamily classification, the protein sequences of all TaRRs were further analyzed for conserved domains, including the receiver domain, Myb-like DNA binding domain of RRBs, and CCT domain of PRRs. Among the 151 *TaRR* genes, there were 20 *TaRRAs*, 71 *TaRRBs*, 43 *TaRRCs*, and 17 *TaPRRs* (Supplementary Table 2), and each type of *TaRR* gene in wheat was found to be more abundant than the corresponding type of *RR* genes in *Arabidopsis* and rice (Table 1).

**TABLE 1:** Summary of the RR superfamily in wheat, *Arabidopsis*, and rice.

Classification	Wheat	*Arabidopsis*	Rice
RRA	20	10	13
RRB	71	14	16
RRC	43	2	2
PRR	17	5	5
RR (for potential new clades)	0	1	0
Total	151	32	36

**FIGURE 2:**
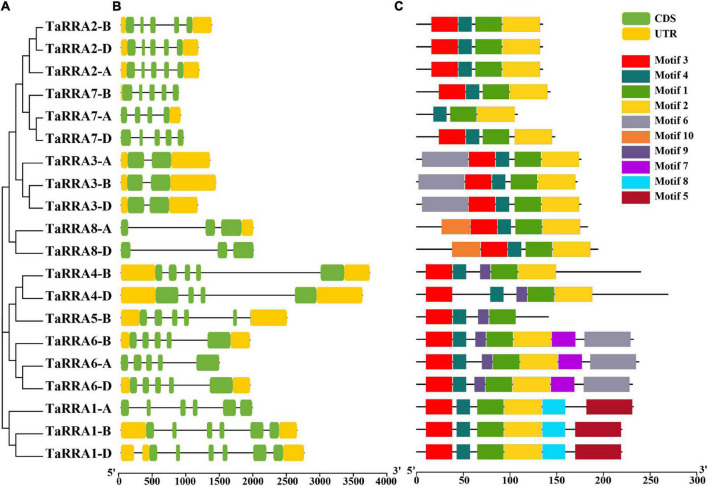
Phylogenetic analysis, gene structure, and conserved motifs of TaRRAs. **(A)** The phylogenetic tree was constructed based on the full-length amino acid sequence of TaRRAs. **(B)** Gene structure analysis of *TaRRA* genes. Yellow boxes represent UTR regions; green boxes indicate exons; black lines represent introns. **(C)** Conserved domain structures identified in the TaRRAs. Different color boxes show different motifs.

Further phylogenetic analysis using the full-length protein sequence of type-A RRs in wheat clustered the 20 *TaRRA* genes into eight homologous groups (Figure 2A), which were named *TaRRA1* to *TaRRA8*. *TaRRA1, 2, 3, 6*, and *7* had three orthologous genes (*TaRRA1-A/B/D, TaRRA2-A/B/D, TaRRA3-A/B/D, TaRRA6-A/B/D*, and *TaRRA7-A/B/D*), while *TaRRA4* and *8* contained two orthologous genes (*TaRRA4-B/D* and *TaRRA8-A/D*) and *TaRRA5* possessed only one gene copy (*TaRRA5-B*). All the proteins encoded by *TaRRA* genes varied from 108 to 269 amino acids with predicted molecular weights (MW) ranging from 12.22 to 28.89 kDa and the isoelectric points (PI) ranging from 5.05 to 8.48 (Table 2).

**TABLE 2:** Information of the *TaRRA* gene members in wheat.

Gene name	ID	Chromosome location	Exon	cDNA (bp)	Protein (aa)	MW (kDa)	PI
*TaRRA1-A*	TraesCS2A02G309700.1	2A:532865797-532867753	6	699	232	25.49	6.74
*TaRRA1-B*	TraesCS2B02G326800.2	2B:467870720-467873343	6	1,305	220	24.09	8.48
*TaRRA1-D*	TraesCS2D02G308000.1	2D:394798248-394800977	6	1,276	220	24.06	8.48
*TaRRA2-A*	TraesCS2A02G377900.1	2A:620212372-620213537	5	728	135	14.72	7.77
*TaRRA2-B*	TraesCS2B02G394900.1	2B:559553430-559554782	5	768	135	14.72	7.77
*TaRRA2-D*	TraesCS2D02G374300.1	2D:477627271-477628424	5	735	135	14.72	7.77
*TaRRA3-A*	TraesCS2A02G551100.1	2A:758102501-758103826	2	1,210	176	19.12	5.90
*TaRRA3-B*	TraesCS2B02G582300.1	2B:769949352-769950767	2	1,293	172	18.72	5.50
*TaRRA3-D*	TraesCS2D02G552200.1	2D:627582838-627583982	2	1,061	176	19.12	5.90
*TaRRA4-B*	TraesCS3B02G548600.1	3B:784040014-784043723	5	1,616	240	25.90	5.33
*TaRRA4-D*	TraesCS3D02G494600.1	3D:587200633-587204237	4	2,013	269	28.89	5.88
*TaRRA5-B*	TraesCS4B02G178900.1	4B:392001595-392004069	6	1,248	141	15.36	5.55
*TaRRA6-A*	TraesCS5A02G131600.2	5A:296468274-296469739	5	717	238	26.24	5.61
*TaRRA6-B*	TraesCS5B02G132900.2	5B:247665062-247666984	5	1,116	232	25.36	5.14
*TaRRA6-D*	TraesCS5D02G140200.1	5D:223887585-223889509	5	1,088	231	25.36	5.05
*TaRRA7-A*	TraesCS6A02G221800.1	6A:412848604-412849492	4	493	108	12.22	6.58
*TaRRA7-B*	TraesCS6B02G259700.1	6B:469707108-469707967	5	451	143	15.39	7.88
*TaRRA7-D*	TraesCS6D02G213700.2	6D:303981610-303982542	5	447	148	15.91	5.85
*TaRRA8-A*	TraesCS6A02G416400.1	6A:615462243-615464213	3	727	183	20.10	6.31
*TaRRA8-D*	TraesCS6D02G401600.1	6D:470632933-470634909	3	585	194	21.29	8.45

### Gene structure and conserved motifs of *TaRRAs*

To gain further insights into the *TaRRA* gene members, we surveyed the gene structure and conserved motifs of each *TaRRA*. Although the lengths of genomic DNA varied from 859 to 3,709 bp in different *TaRRAs*, orthologous genes at each *TaRRA* locus usually had similar genomic DNA lengths. For instance, genomic DNA length of *TaRRA7-A, TaRRA7-B*, and *TaRRA7-D* was 888, 859, and 932 bp, respectively, while genomic DNA length of *TaRRA4-B* and *TaRRA4-D* was 3,709 and 3,604 bp, respectively (Figure 2B). Additionally, the number of exons, ranging from 2 to 6 in different *TaRRAs*, was generally the same in orthologous genes at each *TaRRA* locus. For example, *TaRRA3-A, TaRRA3-B*, and *TaRRA3-D* all harbored 2 exons, while *TaRRA1-A, TaRRA1-B*, and *TaRRA1-D* all contained 6 exons (Figure 2B and Table 2).

Using the MEME tool to predict the conserved protein motifs of the TaRRA family, 10 conversed motifs were identified (Figure 2C and Supplementary Table 3). Except for TaRRA5-B and TaRRA7-A, all TaRRAs contained motif 1, motif 2, motif 3, and motif 4. Additionally, TaRRA1-A, TaRRA1-B, and TaRRA1-D also possessed motif 5 and motif 8 (both unique in this group). TaRRA3-A, TaRRA3-B, and TaRRA3-D also had motif 6, while TaRRA4-B and TaRRA4-D had an added motif 9. TaRRA6-A, TaRRA6-B, and TaRRA6-D additionally contained motif 6, motif 7 (unique in this group), and motif 9. Likewise, TaRRA8-A and TaRRA8-D had motif 10 (unique in this group). In addition, all TaRRAs contained the highly conserved Lys and two Asp residues (D-D-K) in the receiver domain, except for TaRRA5-B and TaRRA7-A, which lacked the Lys and the first Asp respectively (Supplementary Figure 1). It is worth mentioning that all the 20 TaRRAs contained the predicted Asp phosphorylation site (the second Asp in the conserved D-D-K motif), which was embedded in a conserved TDY sequence. The above results indicate that orthologous *TaRRA* genes in the A, B, and D wheat subgenomes were usually similar in gene structure and encoding protein motifs, suggesting that *TaRRA* genes were conserved during evolution.

### Chromosomal distribution and synteny analysis of *TaRRAs*

**FIGURE 3:**
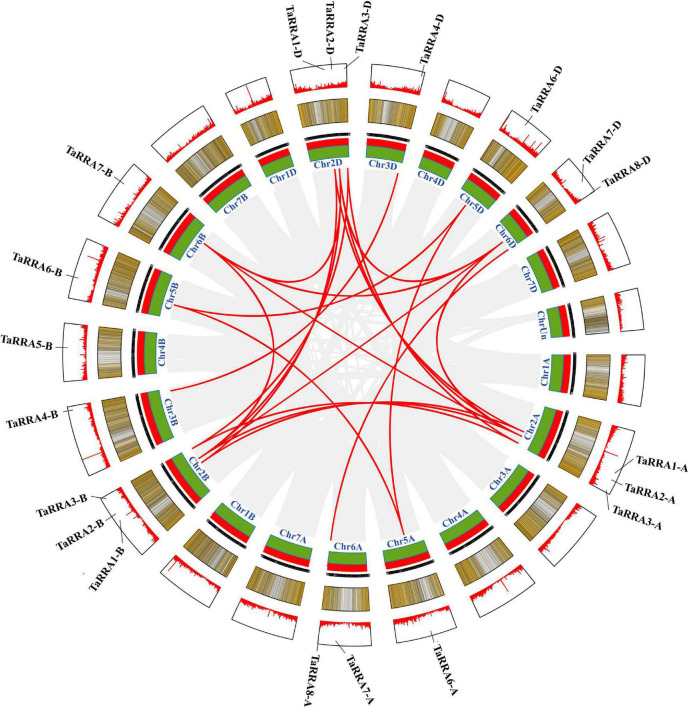
Schematic diagram of the inter-chromosomal relationships of *TaRRA* genes. Gray lines indicate all syntenic blocks in the wheat genome, and red lines indicate the presence of *TaRRA* genes.

Chromosomal localization analysis showed that the 20 *TaRRA* genes were unevenly distributed on 12 of the 21 wheat chromosomes (Figure 3), with the number of *TaRRA* genes on each chromosome ranging from 1 (3B, 3D, 4B, 5A, 5B, 5D, and 6B) to 3 (2A, 2B, and 2D). Chromosomal group II harbored 9 (45.0%) *TaRRA* genes, the largest number, followed by chromosomal groups VI, V, III, and IV, which contained 5 (25.0%), 3 (15.0%), 2 (10.0%), and 1 (5.0%) *TaRRA* genes, respectively (Figure 3). Whereas, there was no *TaRRA* gene located in the rest two chromosomal groups (I and VII). Collinear relationship displayed the homology between *TaRRAs* (Figure 3), which was consistent with the phylogenetic analysis (Figure 2).

**FIGURE 4:**
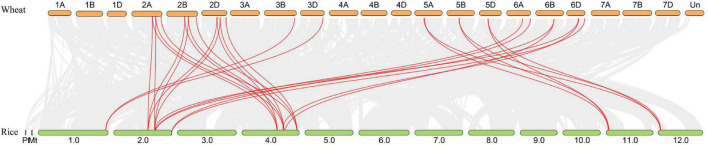
Synteny analysis of *RRA* genes in wheat and rice. Gray lines in the background indicate all syntenic blocks within the two genomes, while red lines highlight the syntenic *RRA* gene pairs.

Synteny analysis between the wheat and rice was conducted and 30 orthologous *RRA* gene pairs were found (Figure 4 and Supplementary Table 4), with a close similarity to the phylogenetic analysis (Figure 1), indicating that these syntenic gene pairs were relatively conserved during the evolution of gramineous species.

### Prediction of *Cis*-acting regulatory elements in the promoter of *TaRRAs*

**FIGURE 5:**
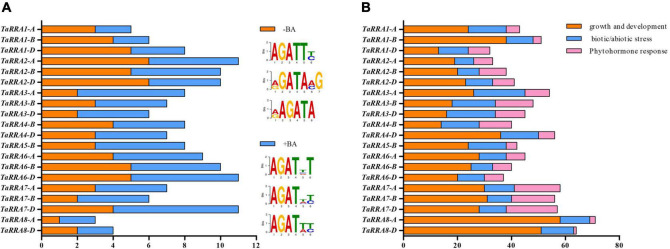
Prediction of *cis*-acting elements in the promoter region of *TaRRAs.* **(A)** BA-dependent (+BA) and BA-independent (-BA) RRB binding *cis*-acting elements in the promoter of *TaRRAs*. **(B)** Growth and development, biotic/abiotic stress, and phytohormone responses *cis*-acting elements in the promoter of *TaRRAs*.

The *cis*-acting elements in the promoter region play important roles in gene transcription regulation. The *RRAs* are cytokinin response genes that are targets of RRB transcription factors in *Arabidopsis* (To et al., 2004). Therefore, the 1,500 bp DNA sequence upstream of *TaRRAs* was analyzed for BA-dependent (+BA) and BA-independent (-BA) RRB binding *cis*-acting elements (Figure 5A and Supplementary Table 5; Xie et al., 2018). Both the BA-dependent and BA-independent RRB binding *cis*-acting elements were distributed widely throughout all of the *TaRRA* genes, suggesting that transcription of *TaRRAs* was probably regulated in part by RRBs.

To fully understand the potential role of *TaRRAs*, the 1,500 bp promoter region of *TaRRAs* was further analyzed in the PlantCARE database for more *cis*-acting elements. A total of 43 types of *cis*-acting elements with known functions were identified in the *TaRRAs* promoter region, which were divided into three different categories, i.e., growth and development response elements, biotic/abiotic stress response elements, and phytohormone response elements (Figure 5B and Supplementary Tables 6, 7). Among the growth and development response *cis*-elements, CAAT-box (common *cis*-acting element in promoter and enhancer regions) and TATA-box (core promoter element around -30 of transcription start) were highly enriched in all the *TaRRA* promoters. In addition, O2-site involved in zein metabolism regulation, RY-element involved in seed-specific regulation, and CAT-box related to meristem expression were identified in some of the *TaRRA* promoters. Among the biotic/abiotic stress response *cis*-elements, the proportion of light-response elements was large, including 19 *cis*-regulatory factors, such as G-box, Sp1, and TCCC-motif. Additionally, ARE and GC-motif involved in anaerobic induction and LTR and MBS elements involved in low-temperature and drought responsiveness, respectively, were found in several *TaRRA* promoters. In the phytohormone response category, ABRE in ABA response and CGTCA-motif and TGACG-motif in MeJA response were distributed in most of the *TaRRA* promoters. Moreover, GARE-motif, P-box, and TATC-box implicated in gibberellin response, AuxRR-core and TGA-element in auxin response, and TCA-element in salicylic acid response were also distributed in several *TaRRA* promoters. The presence of different numbers and types of *cis*-acting elements in *TaRRA* promoters indicates that these genes may be involved in different regulatory mechanisms.

### Expression pattern of *TaRRAs* in different tissues and response to cytokinin

**FIGURE 6:**
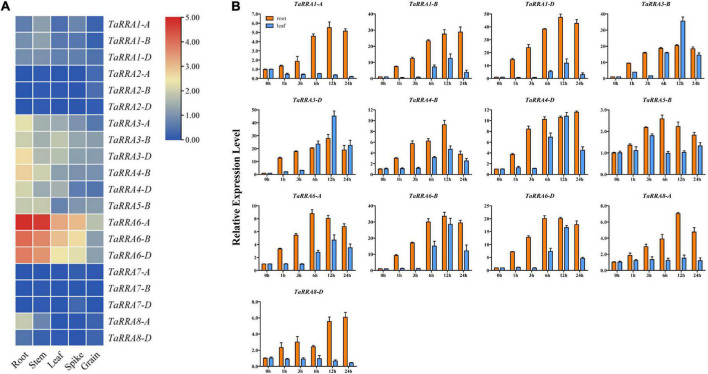
Expression analysis of *TaRRA* genes. **(A)** Expression profiles of *TaRRA* genes in various tissues. The RNA-sequence data was obtained from leaf, root, stem, spike, and grain of Chinese Spring (International Wheat Genome Sequencing Consortium [IWGSC], 2014). **(B)** Quantitative RT-PCR analysis of *TaRRA* genes in response to cytokinin treatment. Leaves and roots of 10-day-old seedlings were sampled after 50 μM 6-BA treatment for 0, 1, 3, 6, 12, and 24 h. The data are given as means ± SE of three biological replicates.

To characterize the expression profiles of the *TaRRA* gene family, we analyzed the RNA-seq data downloaded from the WheatOmics 1.0 (Ma et al., 2021). Notably, *TaRRA2-A/B/D* and *TaRRA7-A/B/D* were hardly detected in any of the tissues tested, while other *TaRRAs* showed relatively high expression in the root, except for *TaRRA1-A/B/D* with higher expression in the stem (Figure 6A).

Since most of the *RRAs* are rapidly induced by exogenous cytokinin in monocots and dicots (D’Agostino et al., 2000; Jain et al., 2006), we investigated the expression profiles of *TaRRAs* in response to cytokinin treatment by qRT-PCR (Figure 6B). In the root, 10 out of 13 detectable *TaRRAs* were up-regulated after 1 h BA treatment and displayed an increasing expression trend afterward. Most of the *TaRRAs* reached maximal induction at 12 h, except for *TaRRA5-B* and *TaRRA6-A*, which were maximally induced at 6 h, and *TaRRA1-B, TaRRA4-D*, and *TaRRA8-D*, which were maximally induced at 24 h. Although all the 13 detectable *TaRRAs* were cytokinin-induced, their fold-change varied greatly after BA treatment, ranging from 2.6 (*TaRRA5-B*, 6 h) to 47.3 (*TaRRA1-D*, 12 h) times compared with the control (0 h). It is worth noting that expression levels of orthologous genes at each *TaRRA* locus could differ significantly after BA treatment. For instance, *TaRRA1-D* was up-regulated by 47.3 times at 12 h, whereas *TaRRA1-A* was up-regulated by 5.5 times. In the leaf, 9 out of 13 detectable *TaRRAs* displayed an obvious increase in the transcription level after 6 h BA treatment and showed maximal induction at 12 h. However, there was no increase in the transcription levels of *TaRRA5-B* and *TaRRA8-A*, and a decrease was observed in the transcription levels of *TaRRA1-A* and *TaRRA8-D*. The above results showed that most *TaRRAs* responded more rapidly and strongly to BA treatment in the root than that in the leaf. However, we could hardly detect the expression of *TaRRA2-A/B/D* and *TaRRA7-A/B/D* by qRT-PCR, which was consistent with previous RNA-seq data (Figure 6A).

### Expression pattern of *TaRRAs* under different stresses

**FIGURE 7:**
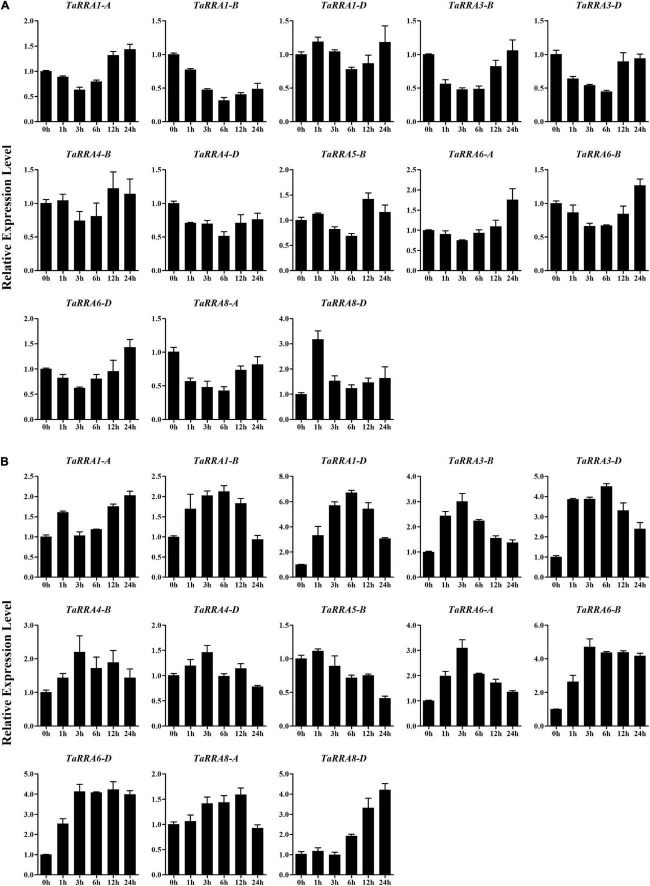
Quantitative RT-PCR analysis of *TaRRA* genes under drought and salt stress. Roots of 10-day-old seedlings were sampled after 0, 1, 3, 6, 12, and 24 h drought stress **(A)** and salt stress **(B)**. The data are given as means ± SE of three biological replicates.

**FIGURE 8:**
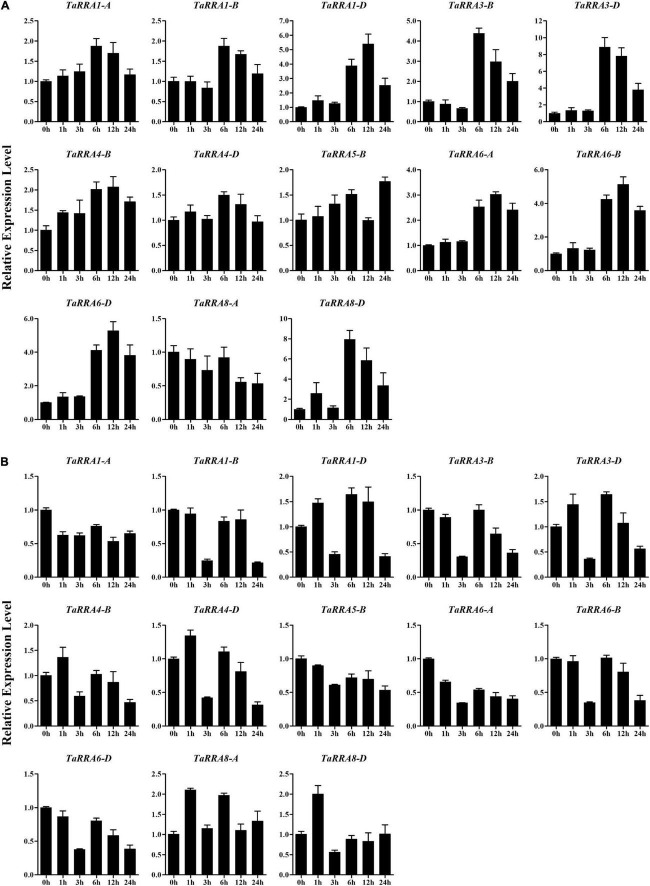
Quantitative RT-PCR analysis of *TaRRA* genes under cold and heat stress. Roots of 10-day-old seedlings were sampled after 0, 1, 3, 6, 12, and 24 h cold stress **(A)** and heat stress **(B)**. The data are given as means ± SE of three biological replicates.

To further evaluate the potential function of *TaRRAs* in response to abiotic stress, the *TaRRA* gene expression patterns were analyzed by qRT-PCR under drought, salt, cold, and heat stress treatments (Figures 7, 8). The results showed that under drought stress, the expression levels of *TaRRA1-B, TaRRA3-B, TaRRA3-D, TaRRA4-D*, and *TaRRA8-A* decreased continuously from 0 to 6 h and later increased marginally at 12 and 24 h (Figure 7A). *TaRRA8-D* was exclusively up-regulated by drought stress, while the expression of the rest of the *TaRRAs* showed insignificant changes under drought stress. Under salt stress, the expression levels of most *TaRRAs* were up-regulated at least at one time point (Figure 7B). Notably, *TaRRA1-D, TaRRA3-D, TaRRA6-B*, and *TaRRA6-D* were induced at each time point compared with the control (0 h). Inversely, *TaRRA5-B* was gradually down-regulated by salt stress. Under cold stress, the expression of 8 *TaRRAs* increased after 6 h treatment, among which the expression of *TaRRA1-D, TaRRA4-B, TaRRA6-A, TaRRA6-B*, and *TaRRA6-D* continued to increase till 12 h, whereas the expression of *TaRRA3-B, TaRRA3-D*, and *TaRRA8-D* was reduced subsequently (Figure 8A). Heat significantly inhibited the expression of most *TaRRAs* after 3 h treatment, except for that of *TaRRA8-A* and *TaRRA8-D*, which increased after 1 h treatment (Figure 8B).

It is worth noting that some orthologous genes at each *TaRRA* locus (*TaRRA3-B/D* and *TaRRA6-A/B/D*) exhibited similar expression patterns in response to abiotic stress, indicating that they may have similar biological functions under stress conditions. On the contrary, *TaRRA8-A* and *TaRRA8-D* showed different expression patterns under drought and cold stress, suggesting that they could play different roles under some abiotic stresses. In general, all the *TaRRA*s showed significant changes in response to at least one abiotic stress.

## Discussion

*RRAs* are rapidly induced by exogenous cytokinin and are thus considered to be primary cytokinin response genes (D’Agostino et al., 2000; Jain et al., 2006). The *RRA* gene family, a subfamily of the *RR* gene family, is relatively small in higher plants, with only 10, 13, 21, and 18 members in *Arabidopsis*, rice, maize, and soybean, respectively (Mochida et al., 2010; Chu et al., 2011; Heyl et al., 2013). In this study, we performed a systematic genome-wide analysis of the wheat *RRA* gene family by combining two different approaches. First, we performed phylogenetic analysis by using the conserved receiver domain of 151 TaRRs, and simultaneously adding the well-established family members from *Arabidopsis* and rice into the analysis (Figure 1; Heyl et al., 2013). Second, we analyzed the conserved domains of all the TaRRs to further confirm whether they contain a Myb-like DNA binding domain or a CCT domain in addition to the receiver domain. We used the protein sequence of the longest transcript of each TaRR for domain analysis to avoid domain missing of the shorter transcript. According to the above method, we identified 20 *RRAs*, 71 *RRBs*, 43 *RRCs*, and 17 *PRRs* from the wheat reference genome (Table 1). However, in a previous study, 41 *RRAs*, 2 *RRBs*, and 2 *PRRs* have been identified in wheat (Gahlaut et al., 2014). Given that RRAs only carry a receiver domain, while RRBs contain an additional Myb-like DNA binding domain and PRRs have an extra CCT domain, some truncated RRBs and PRRs may be identified as RRAs due to the earlier incomplete wheat reference genome. In recent years, great progress has been made in genome sequencing, assembly, and annotation of wheat (International Wheat Genome Sequencing Consortium [IWGSC], 2018; Alonge et al., 2020; Zhu et al., 2021), which provides a high-quality reference genome for the study of the *TaRRA* gene family. The 20 *TaRRA* genes belonging to 8 homologous groups were unequally distributed on 12 wheat chromosomes (Figure 3). As wheat is an allohexaploid (AABBDD), most *TaRRA* genes had homologs in the A, B, and D subgenomes due to polyploidization, sharing similar gene structures and protein motifs within the homologous group (Figure 2).

Most of the *RRAs* are rapidly induced by exogenous cytokinin in plants (D’Agostino et al., 2000; Asakura et al., 2003; Jain et al., 2006). Consistent with previous results, the transcription levels of most detectable *TaRRAs* displayed an obvious increase after BA treatment in both the root and leaf (Figure 6B). However, the transcripts of most *TaRRAs* reached a maximal induction at 12 h cytokinin treatment, while most *RRAs* in *Arabidopsis* and rice showed maximal induction within 1 h cytokinin treatment and then gradually declined (D’Agostino et al., 2000; Jain et al., 2006). Although *TaRRA1-A, TaRRA5-B, TaRRA8-A*, and *TaRRA8-D* were induced by cytokinin in the root, their transcription levels were unchanged or even decreased in the leaf after cytokinin treatment (Figure 6B), indicating their potential function differentiation between root and leaf. Similarly, there is no significant change in the transcript abundance of a few *RRAs* in *Arabidopsis* and rice following cytokinin treatment, including *ARR8, ARR9, OsRR3*, and *OsRR8* (D’Agostino et al., 2000; Jain et al., 2006). The rapid induction of *RRAs* by exogenous cytokinin has been shown to mediate a feedback mechanism, probably by competing with RRBs for the phosphotransfer from HPs. Phosphorylation of RRAs increases their protein stability (To et al., 2007), whereas phosphorylation of RRBs enables them to bind to DNA, thus initiating transcription of downstream targets (Kim et al., 2006; Zubo et al., 2017). Double and higher-order type-A *arr* mutants, rather than the single *arr* mutants, show increasing sensitivity to cytokinin, indicating that RRAs act as negative regulators of cytokinin signaling with partially redundant functions (To et al., 2007).

Cytokinin signaling components are widely involved in plant response to abiotic stress. A series of *Arabidopsis* mutants, including *ahk2,3, ahp2,3,5*, and *arr1,10,12*, show significantly increased drought and salt tolerance, indicating that HKs, HPs, and RRBs are negative regulators of these stress responses (Tran et al., 2007; Nishiyama et al., 2013; Nguyen et al., 2016; Abdelrahman et al., 2021). In contrast, plants overexpressing *ARR5* exhibit enhanced drought tolerance, suggesting that type-A ARR5 is a positive regulator of drought tolerance (Huang et al., 2018). Functional analysis of the single and double *ahk* mutants indicates that HKs function partially redundantly as negative regulators of the cold stress adaptation response (Jeon et al., 2010). However, type-B ARR1 is a positive factor in cold signaling, because *arr1* shows reduced cold resistance, whereas *ARR1* overexpression increases plant cold resistance (Jeon and Kim, 2013). Additionally, RRAs also play important roles in cold stress signaling (Jeon et al., 2010; Shi et al., 2012).

Given that the expression pattern of genes has a correlation with its function, we monitored the expression profiles of *TaRRA* family members under multiple stresses. Drought stress reduced the expression of *TaRRA1-B, TaRRA3-B, TaRRA3-D, TaRRA4-D*, and *TaRRA8-A*, but induced the expression of *TaRRA8-D* (Figure 7A). Similarly, drought stress reduces the expression of *ARR8* and *ARR17*, whereas induces the expression of *ARR5, ARR7*, and *ARR15* in *Arabidopsis* (Kang et al., 2012). We identified 8 *TaRRAs* with significantly up-regulated expression under cold stress (Figure 8A), which is consistent with the cold-induced changes in *ARR5, ARR6, ARR7*, and *ARR15* expression in *Arabidopsis* (Jeon et al., 2010). Furthermore, overexpression of *ARR5, ARR7*, and *ARR15* enhances the freezing tolerance of plants, while *arr5, arr6*, and *arr7* also lead to higher freezing tolerance (Jeon et al., 2010; Shi et al., 2012). These different results suggest the complexity of the molecular mechanism involved and hence further research is warranted. In addition, a considerable number of *TaRRAs* were shown to respond to salt and heat stress (Figures 7B, 8B), suggesting that they are promising regulators for salt and heat response in wheat. *TaRRA3-B/D*, orthologs of drought and salt positive regulator *OsRR6*, and *TaRRA6-A/B/D*, orthologs of salt negative regulator *OsRR9* and *OsRR10* (Supplementary Table 4), showed responses to salt, cold, and heat stress, which may be important candidate genes for genetic improvement of stress tolerance in wheat.

In conclusion, this study provided comprehensive insights into the *TaRRA* gene family in wheat. The systematical identification and investigation of the *TaRRA* gene family will inevitably contribute to further elucidation of the biological function and genetic improvement application of *TaRRAs* in wheat.

## Data availability statement

The original contributions presented in this study are included in the article/Supplementary material, further inquiries can be directed to the corresponding authors.

## Author contributions

HL and YJZ designed the experiments. LS, LL, JZ, MH, YLZ, YZ, XT, PW, QL, and XC performed the experiments and analyzed the data. LS and LL wrote the manuscript. All authors reviewed and approved the manuscript.

## Footnotes

1
https://plants.ensembl.org/index.html

2
https://pfam.xfam.org

3
http://smart.embl.de/

4
https://www.ncbi.nlm.nih.gov/Structure/cdd/wrpsb.cgi

5
https://web.expasy.org/compute_pi/

6
http://gsds.gao-lab.org/

7
https://meme-suite.org/meme/tools/meme

8
https://github.com/CJ-Chen/TBtools

9
http://bioinformatics.psb.ugent.be/webtools/plantcare/html/

## References

[B1] Abdelrahman M. Nishiyama R. Tran C. D. Kusano M. Nakabayashi R. Okazaki Y. (2021). Defective cytokinin signaling reprograms lipid and flavonoid gene-to-metabolite networks to mitigate high salinity in *Arabidopsis*. *Proc. Natl. Acad. Sci. U.S.A.* 118:e2105021118. 10.1073/pnas.2105021118 34815339 PMC8640937

[B2] Alonge M. Shumate A. Puiu D. Zimin A. V. Salzberg S. L. (2020). Chromosome-scale assembly of the bread wheat genome reveals thousands of additional gene copies. *Genetics* 216 599–608. 10.1534/genetics.120.303501 32796007 PMC7536849

[B3] Argueso C. T. Raines T. Kieber J. J. (2010). Cytokinin signaling and transcriptional networks. *Curr. Opin. Plant Biol.* 13 533–539. 10.1016/j.pbi.2010.08.006 20851038

[B4] Argyros R. D. Mathews D. E. Chiang Y. H. Palmer C. M. Thibault D. M. Etheridge N. (2008). Type B response regulators of *Arabidopsis* play key roles in cytokinin signaling and plant development. *Plant Cell* 20 2102–2116. 10.1105/tpc.108.059584 18723577 PMC2553617

[B5] Asakura Y. Hagino T. Ohta Y. Aoki K. Yonekura-Sakakibara K. Deji A. (2003). Molecular characterization of His-Asp phosphorelay signaling factors in maize leaves: Implications of the signal divergence by cytokinin-inducible response regulators in the cytosol and the nuclei. *Plant Mol. Biol.* 52 331–341. 10.1023/a:1023971315108 12856940

[B6] Bhaskar A. Paul L. K. Sharma E. Jha S. Jain M. Khurana J. P. (2021). OsRR6, a type-A response regulator in rice, mediates cytokinin, light and stress responses when over-expressed in *Arabidopsis*. *Plant Physiol. Biochem.* 161 98–112. 10.1016/j.plaphy.2021.01.047 33581623

[B7] Chen C. Chen H. Zhang Y. Thomas H. R. Frank M. H. He Y. (2020). TBtools: An integrative toolkit developed for interactive analyses of big biological data. *Mol. Plant* 13 1194–1202. 10.1016/j.molp.2020.06.009 32585190

[B8] Chu Z. X. Ma Q. Lin Y. X. Tang X. L. Zhou Y. Q. Zhu S. W. (2011). Genome-wide identification, classification, and analysis of two-component signal system genes in maize. *Genet. Mol. Res.* 10 3316–3330. 10.4238/2011.December.8.3 22194197

[B9] D’Agostino I. B. Deruère J. Kieber J. J. (2000). Characterization of the response of the *Arabidopsis* response regulator gene family to cytokinin. *Plant Physiol.* 124 1706–1717. 10.1104/pp.124.4.1706 11115887 PMC59868

[B10] El-Showk S. Ruonala R. Helariutta Y. (2013). Crossing paths: Cytokinin signalling and crosstalk. *Development* 140 1373–1383. 10.1242/dev.086371 23482484

[B11] Gahlaut V. Mathur S. Dhariwal R. Khurana J. P. Tyagi A. K. Balyan H. S. (2014). A multi-step phosphorelay two-component system impacts on tolerance against dehydration stress in common wheat. *Funct. Integr. Genomics* 14 707–716. 10.1007/s10142-014-0398-8 25228409

[B12] Ha S. Vankova R. Yamaguchi-Shinozaki K. Shinozaki K. Tran L. S. (2012). Cytokinins: Metabolism and function in plant adaptation to environmental stresses. *Trends Plant Sci.* 17 172–179. 10.1016/j.tplants.2011.12.005 22236698

[B13] Heyl A. Brault M. Frugier F. Kuderova A. Lindner A. C. Motyka V. (2013). Nomenclature for members of the two-component signaling pathway of plants. *Plant Physiol.* 161 1063–1065. 10.1104/pp.112.213207 23324541 PMC3585578

[B14] Hosoda K. Imamura A. Katoh E. Hatta T. Tachiki M. Yamada H. (2002). Molecular structure of the GARP family of plant Myb-related DNA binding motifs of the *Arabidopsis* response regulators. *Plant Cell* 14 2015–2029. 10.1105/tpc.002733 12215502 PMC150752

[B15] Huang X. Hou L. Meng J. You H. Li Z. Gong Z. (2018). The antagonistic action of abscisic acid and cytokinin signaling mediates drought stress response in *Arabidopsis*. *Mol. Plant* 11 970–982. 10.1016/j.molp.2018.05.001 29753021

[B16] Hwang I. Sheen J. Müller B. (2012). Cytokinin signaling networks. *Annu. Rev. Plant Biol.* 63 353–380. 10.1146/annurev-arplant-042811-105503 22554243

[B17] International Wheat Genome Sequencing Consortium [IWGSC] (2014). A chromosome-based draft sequence of the hexaploid bread wheat (*Triticum aestivum*) genome. *Science* 345:1251788. 10.1126/science.1251788 25035500

[B18] International Wheat Genome Sequencing Consortium [IWGSC] (2018). Shifting the limits in wheat research and breeding using a fully annotated reference genome. *Science* 361:eaar7191. 10.1126/science.aar7191 30115783

[B19] Jain M. Tyagi A. K. Khurana J. P. (2006). Molecular characterization and differential expression of cytokinin-responsive type-A response regulators in rice (*Oryza sativa*). *BMC Plant Biol.* 6:1. 10.1186/1471-2229-6-1 16472405 PMC1382228

[B20] Jeon J. Kim J. (2013). *Arabidopsis* response regulator1 and *Arabidopsis* histidine phosphotransfer protein2 (AHP2), AHP3, and AHP5 function in cold signaling. *Plant Physiol.* 161 408–424. 10.1104/pp.112.207621 23124324 PMC3532271

[B21] Jeon J. Kim N. Y. Kim S. Kang N. Y. Novák O. Ku S. J. (2010). A subset of cytokinin two-component signaling system plays a role in cold temperature stress response in *Arabidopsis*. *J. Biol. Chem.* 285 23371–23386. 10.1074/jbc.M109.096644 20463025 PMC2906329

[B22] Kang N. Y. Cho C. Kim N. Y. Kim J. (2012). Cytokinin receptor-dependent and receptor-independent pathways in the dehydration response of *Arabidopsis thaliana*. *J. Plant Physiol.* 169 1382–1391. 10.1016/j.jplph.2012.05.007 22704545

[B23] Kieber J. J. Schaller G. E. (2018). Cytokinin signaling in plant development. *Development* 145:dev149344. 10.1242/dev.149344 29487105

[B24] Kim H. J. Ryu H. Hong S. H. Woo H. R. Lim P. O. Lee I. C. (2006). Cytokinin-mediated control of leaf longevity by AHK3 through phosphorylation of ARR2 in *Arabidopsis*. *Proc. Natl. Acad. Sci. U.S.A.* 103 814–819. 10.1073/pnas.0505150103 16407152 PMC1334631

[B25] Kumar S. Stecher G. Tamura K. (2016). MEGA7: Molecular evolutionary genetics analysis version 7.0 for bigger datasets. *Mol. Biol. Evol.* 33 1870–1874. 10.1093/molbev/msw054 27004904 PMC8210823

[B26] Larkin M. A. Blackshields G. Brown N. P. Chenna R. McGettigan P. A. McWilliam H. (2007). Clustal W and Clustal X version 2.0. *Bioinformatics* 23 2947–2948. 10.1093/bioinformatics/btm404 17846036

[B27] Leibfried A. To J. P. Busch W. Stehling S. Kehle A. Demar M. (2005). WUSCHEL controls meristem function by direct regulation of cytokinin-inducible response regulators. *Nature* 438 1172–1175. 10.1038/nature04270 16372013

[B28] Liu Y. Zhang M. Meng Z. Wang B. Chen M. (2020). Research progress on the roles of cytokinin in plant response to stress. *Int. J. Mol. Sci.* 21:6574. 10.3390/ijms21186574 32911801 PMC7555750

[B29] Ma S. Wang M. Wu J. Guo W. Chen Y. Li G. (2021). WheatOmics: A platform combining multiple omics data to accelerate functional genomics studies in wheat. *Mol. Plant* 14 1965–1968. 10.1016/j.molp.2021.10.006 34715393

[B30] Makino S. Kiba T. Imamura A. Hanaki N. Nakamura A. Suzuki T. (2000). Genes encoding pseudo-response regulators: Insight into His-to-Asp phosphorelay and circadian rhythm in *Arabidopsis thaliana*. *Plant Cell Physiol.* 41 791–803. 10.1093/pcp/41.6.791 10945350

[B31] Mira-Rodado V. Sweere U. Grefen C. Kunkel T. Fejes E. Nagy F. (2007). Functional cross-talk between two-component and phytochrome B signal transduction in *Arabidopsis*. *J. Exp. Bot.* 58 2595–2607. 10.1093/jxb/erm087 17545225

[B32] Mochida K. Yoshida T. Sakurai T. Yamaguchi-Shinozaki K. Shinozaki K. Tran L. S. (2010). Genome-wide analysis of two-component systems and prediction of stress-responsive two-component system members in soybean. *DNA Res.* 17 303–324. 10.1093/dnares/dsq021 20817745 PMC2955714

[B33] Nguyen K. H. Ha C. V. Nishiyama R. Watanabe Y. Leyva-González M. A. Fujita Y. (2016). *Arabidopsis* type B cytokinin response regulators ARR1, ARR10, and ARR12 negatively regulate plant responses to drought. *Proc. Natl. Acad. Sci. U.S.A.* 113 3090–3095. 10.1073/pnas.1600399113 26884175 PMC4801291

[B34] Nishiyama R. Watanabe Y. Leyva-Gonzalez M. A. Van Ha C. Fujita Y. Tanaka M. (2013). *Arabidopsis* AHP2, AHP3, and AHP5 histidine phosphotransfer proteins function as redundant negative regulators of drought stress response. *Proc. Natl. Acad. Sci. U.S.A.* 110 4840–4845. 10.1073/pnas.1302265110 23487796 PMC3606972

[B35] Pavlů J. Novák J. Koukalová V. Luklová M. Brzobohatý B. Černý M. (2018). Cytokinin at the crossroads of abiotic stress signalling pathways. *Int. J. Mol. Sci.* 19:2450. 10.3390/ijms19082450 30126242 PMC6121657

[B36] Sakai H. Aoyama T. Oka A. (2000). *Arabidopsis* ARR1 and ARR2 response regulators operate as transcriptional activators. *Plant J.* 24 703–711. 10.1111/j.1365-313X.2000.00909.x 11135105

[B37] Salomé P. A. To J. P. Kieber J. J. McClung C. R. (2006). *Arabidopsis* response regulators ARR3 and ARR4 play cytokinin-independent roles in the control of circadian period. *Plant Cell* 18 55–69. 10.1105/tpc.105.037994 16326927 PMC1323484

[B38] Schaller G. E. Bishopp A. Kieber J. J. (2015). The yin-yang of hormones: Cytokinin and auxin interactions in plant development. *Plant Cell* 27 44–63. 10.1105/tpc.114.133595 25604447 PMC4330578

[B39] Schaller G. E. Kieber J. J. Shiu S. H. (2008). Two-component signaling elements and histidyl-aspartyl phosphorelays. *Arabidopsis Book* 2008:e0112. 10.1199/tab.0112 22303237 PMC3243373

[B40] Shi Y. Tian S. Hou L. Huang X. Zhang X. Guo H. (2012). Ethylene signaling negatively regulates freezing tolerance by repressing expression of *CBF* and type-A *ARR* genes in *Arabidopsis*. *Plant Cell* 24 2578–2595. 10.1105/tpc.112.098640 22706288 PMC3406918

[B41] Srivastava A. K. Dutta S. Chattopadhyay S. (2019). MYC2 regulates *ARR16*, a component of cytokinin signaling pathways, in *Arabidopsis* seedling development. *Plant Direct* 3:e00177. 10.1002/pld3.177 31788657 PMC6875704

[B42] Sweere U. Eichenberg K. Lohrmann J. Mira-Rodado V. Bäurle I. Kudla J. (2001). Interaction of the response regulator ARR4 with phytochrome B in modulating red light signaling. *Science* 294 1108–1111. 10.1126/science.1065022 11691995

[B43] To J. P. Deruère J. Maxwell B. B. Morris V. F. Hutchison C. E. Ferreira F. J. (2007). Cytokinin regulates type-A *Arabidopsis* response regulator activity and protein stability via two-component phosphorelay. *Plant Cell* 19 3901–3914. 10.1105/tpc.107.052662 18065689 PMC2217641

[B44] To J. P. Haberer G. Ferreira F. J. Deruère J. Mason M. G. Schaller G. E. (2004). Type-A *Arabidopsis* response regulators are partially redundant negative regulators of cytokinin signaling. *Plant Cell* 16 658–671. 10.1105/tpc.018978 14973166 PMC385279

[B45] Tran L. S. Urao T. Qin F. Maruyama K. Kakimoto T. Shinozaki K. (2007). Functional analysis of AHK1/ATHK1 and cytokinin receptor histidine kinases in response to abscisic acid, drought, and salt stress in *Arabidopsis*. *Proc. Natl. Acad. Sci. U.S.A.* 104 20623–20628. 10.1073/pnas.0706547105 18077346 PMC2154481

[B46] Wang W. C. Lin T. C. Kieber J. Tsai Y. C. (2019). Response Regulators 9 and 10 negatively regulate salinity tolerance in rice. *Plant Cell Physiol.* 60 2549–2563. 10.1093/pcp/pcz149 31359043

[B47] Werner T. Schmülling T. (2009). Cytokinin action in plant development. *Curr. Opin. Plant Biol.* 12 527–538. 10.1016/j.pbi.2009.07.002 19740698

[B48] Wybouw B. De Rybel B. (2019). Cytokinin – a developing story. *Trends Plant Sci.* 24 177–185. 10.1016/j.tplants.2018.10.012 30446307

[B49] Xie M. Chen H. Huang L. O’Neil R. C. Shokhirev M. N. Ecker J. R. (2018). A B-ARR-mediated cytokinin transcriptional network directs hormone cross-regulation and shoot development. *Nat. Commun.* 9:1604. 10.1038/s41467-018-03921-6 29686312 PMC5913131

[B50] Zeng R. Li Z. Shi Y. Fu D. Yin P. Cheng J. (2021). Natural variation in a type-A response regulator confers maize chilling tolerance. *Nat. Commun.* 12:4713. 10.1038/s41467-021-25001-y 34354054 PMC8342596

[B51] Zhao Z. Andersen S. U. Ljung K. Dolezal K. Miotk A. Schultheiss S. J. (2010). Hormonal control of the shoot stem-cell niche. *Nature* 465 1089–1092. 10.1038/nature09126 20577215

[B52] Zhu T. Wang L. Rimbert H. Rodriguez J. C. Deal K. R. De Oliveira R. (2021). Optical maps refine the bread wheat *Triticum aestivum* cv. Chinese Spring genome assembly. *Plant J.* 107 303–314. 10.1111/tpj.15289 33893684 PMC8360199

[B53] Zubo Y. O. Blakley I. C. Yamburenko M. V. Worthen J. M. Street I. H. Franco-Zorrilla J. M. (2017). Cytokinin induces genome-wide binding of the type-B response regulator ARR10 to regulate growth and development in *Arabidopsis*. *Proc. Natl. Acad. Sci. U.S.A.* 114 E5995–E6004. 10.1073/pnas.1620749114 28673986 PMC5530654

