# Supplementary material for: Genome-wide identification and expression analysis of the TaRRA gene family in wheat (Triticum aestivum L.)
Source: Front Plant Sci. 2022 Aug 30;13:1006409. doi: 10.3389/fpls.2022.1006409 (PMC9468597; doi:10.3389/fpls.2022.1006409)
Supplement: Supplementary file 1 [file Data_Sheet_1.PDF]

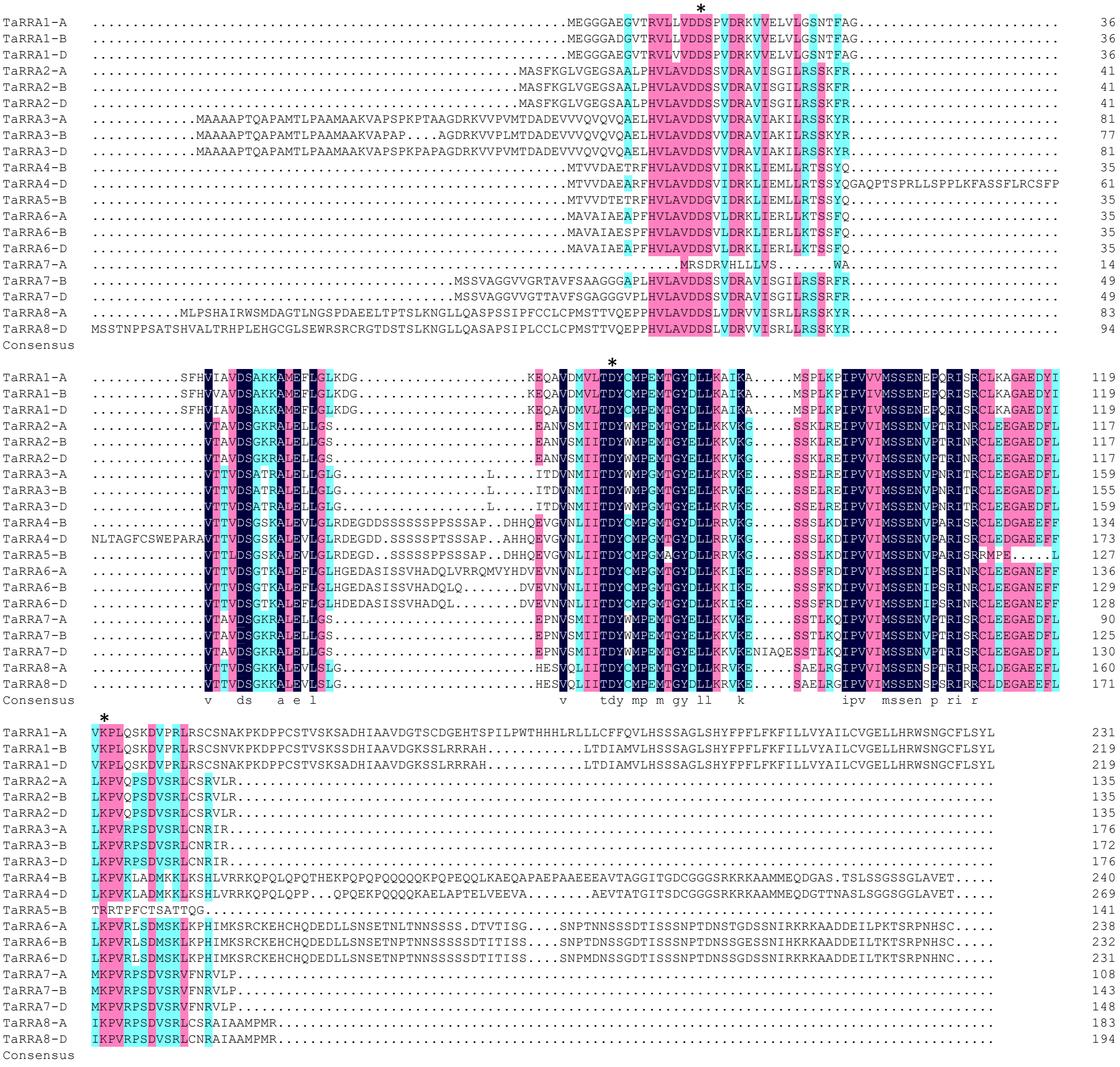

Supplementary Figure 1 | Alignment of RRA amino acid sequences. The highly conserved Lys and two Asp residues (D-D-K) in the receiver domain are marked with an asterisk, including the predicted Asp phosphorylation site, embedded in a conserved TDY sequence.
